# The Electrospray Ionization - Mass Spectra of Erythromycin A Obtained from a Marine *Streptomyces* sp. Mutant

**DOI:** 10.4103/0250-474X.42979

**Published:** 2008

**Authors:** A. M. El-Bondkly, Howaida I. Abd-Alla, M. Shaaban, K. A. Shaaban

**Affiliations:** 1 Applied Microbial Genetics Lab., Genetics and Cytology Dept., National Research Centre, Dokki, Giza 12622, Egypt; 2 Department of Chemistry of Natural Compounds, Division of Pharmaceutical Industries, National Research Centre, El-Behoos st. 33, Dokki-Cairo 12622, Egypt; 3 Department of Organic Chemistry, University of Göttingen, Tammannstrasse 2, D-37077Göttingen, Germany

**Keywords:** Marine *Streptomyces* mutation, bioactivity evaluation, ESI-MS/MS, erythromycin A

## Abstract

In our ongoing search for production improvements of bioactive secondary metabolites from marine *Streptomyces* through the induction of mutations using UV light, out of 145 isolates, mutant 10/14 was able to produce potent antibacterial metabolites other than the parent strain as established by chromatographic analysis. Up-scaling fermentation of mutant 10/14, followed by working up and isolation delivered five metabolites, phenazine, 1-acetyl-*β* -carboline, perlolyrin and erythromycin A, along with an oily substance. The latter two compounds were responsible for the antibacterial activity of the strain. In this article, we discuss with the mutation of the marine *Streptomyces* sp. AH2, bioactivity evaluation, fermentation and isolation of the microbial metabolites. Moreover, we study to first time in detail the 1D and 2D NMR and ESI MS data including ESI MS^2^ and MS^3^ patterns combined with HRESI MS of erythromycin A.

In recent years, marine microorganisms have been given attention as a prodigious source of compounds with intriguing structures and interesting biological activity for drug development. Marine filamentous bacteria, belonging to the genus *Streptomyces* of *Actinomycetes* are an exceptionally rich source for a huge number of secondary metabolites. They are known as one of the most prospective natural sources for production of antibiotics and antitumor compounds^1^–^3^.

Many members of the anthracyclin family are clinically very useful antineoplastic agents with a broad spectrum of activities extending to certain solid tumours that are normally resistant to most other modes of chemotherapy^4^,^5^. The clinical use of such drugs, however, is hampered by a number of undesired side effects, the most serious being the dose-related cardiotoxicity. There is therefore a great interest in related natural or synthetic compounds having improved therapeutic indices^4^–^6^.

Induction of mutations is one of the applied techniques which uses different types of chemical and physical mutagens, either individually or in different combinations, doses and times^7^–^10^. Wieczorek and Mordarski^11^ treated *S. olivaceus* with UV irradiation leading to mutants different from the original strain, not only in their spectrum of antimicrobial activity, but also in the taxonomic properties, e.g. colour of the aerial mycelium, liquefaction of gelatine, growth on cellulose, production of ammonia and nitrate reduction. Lee and Rho^12^ obtained tylosin hyper-producing mutants after treatment of *S. fradiae* NRRL 2702 with either n-nitroso-guanidine (NTG) or exposing to UV. Cheng *et al.*^13^ have varied the productivity of *S. hygroscopicus* FC 904 (the producer of rapamycin) to 60-124% after mutagenesis by NTG and UV. Khattab and EL-Bondkly^14^ used TLC technique to distinguish the produced bioactive components obtained by selected superior mutants compared with the wild type strain. In connection with our search for bioactive components with potential medical application, the marine *Streptomyces* sp. isolate AH2 was subjected to mutation by UV-light. During our biological screening, the most prolific producer of bioactive constituents, mutant 10/14 was selected, upscaled and the bioactive constituents isolated and identified. The potent antibacterial activity of mutant 10/14 was attributed to the major two components, erythromycin A (1) and an oily substance, HM1. We report here the detailed ESI-MS analysis of erythromycin A (1) combined with HRESI-MS along with 2D NMR spectra.

## MATERIALS AND METHODS

NMR spectra were measured on Varian Unity 300 (300.145 MHz) and Varian Inova 600 (599.876 MHz) spectrometers. ESI mass spectra were recorded on a Finnigan LCQ with quaternary pump Rheos 4000 (Flux Instrument). EIMS was performed on a Finnigan MAT95 (70 eV) with perfluorokerosene as reference. IR spectra were recorded on a Perkin-Elmer 1600 Series FT-IR spectrometer (KBr pellets). UV/Vis spectra were recorded on a Perkin-Elmer Lambda 15 UV/VIS spectrometer. Thin layer chromatography (TLC) was performed on Polygram SIL G/UV_254_ (Macherey-Nagel and Co.). *R*_f_ values were measured on Polygram SIL G/UV_254_ (Macherey-Nagel and Co.). Size exclusion chromatography was done on Sephadex LH-20 (Pharmacia).

### Microorganisms and culture conditions:

Marine *Streptomyces* species AH2 was isolated from Suez Canal, Ismailia, Egypt, and was identified according to Bergey^'^s Manual of Systematic Bacteriology 1989^15^. The original marine *Streptomyces* sp. isolate AH2 and their mutants were cultivated at 28° on Trypticase Soy Broth (TSB) medium (Merck Co.) using 75% sea water and 25% distilled water. For seed culture preparation, a loopful of mycelium and spores were taken from the slope culture and used to inoculate 250 ml flask containing 25 ml of TSB medium and then incubated at 28° for 2 d. Five ml of seed culture were used to inoculate 250 ml flasks containing 50 ml TSB medium, and the cultivated flasks were further incubated for additional 5 days at 28° on a rotary shaker (220 rpm). For cultivation of the test bacterial strains (*Escherichia coli* NRRL B-766, *Bacillus subtilis* NRRL B-543 and *Micrococcus luteus* NRRL B-287) Luria Broth (LB) medium was used. It contains (g/l):tryptone (10); yeast extract (5); NaCl (10); Agar (15) and the pH was adjusted to 7.0. Discs used for Antibiotic assay, Whatman product No. 2017, six mm diameter, were saturated with 25 µl supernatant extract of each isolate in comparison with the original strain.

### Mutagenesis:

Marine *Streptomyces* sp. AH2 spores from old slants (7 days) were suspended in sterile distilled water and exposed to UV light (Philips T-UV-30 W lamp type number 57413 p/40) for 5, 10 and 15 min at a distance of 20 cm. After irradiation, the treated suspensions were kept in dark for ~ one hour. Appropriate dilutions were spread on TSB medium and incubated at 28° for 5 d. The growing colonies were transplanted on slants for further studies^14^.

### Fermentation of strain, extraction and separation:

Twenty litres of fermentation medium (TSB) were inoculated with 10% seeding of 10/14 mutant for five days at 28°. After fermentation, the pH of the culture broth had dropped to 3.9. The filtrate was extracted with ethyl acetate, and the organic extract was concentrated in *vacuo* to 500 ml, washed with brine (2×50 ml), then dried over Na_2_SO_4,_ and then evaporated *in vacuo* to dryness. The dark brown extract (748 mg) was flash chromatographed on silica gel of particle size (30-60 μm, 2×70 cm) eluting with an n-hexane/ethyl acetate gradient to deliver five fractions I, *n*-hexane; II, *n*-hexane-EtOAc (9:1); III, *n*-hexane-EtOAc (3:1); IV, *n*-hexane-EtOAc (1:1); V, EtOAc. Purification of the fast fraction I using silica gel (0.014-0.040 μm, 0.8×50 cm) and eluting with *n*-hexane/DCM gradient led to phenazine (2 mg), a greenish-yellow solid. Purification of fraction II on silica gel (0.014-0.040 μm, 0.8×50 cm) eluting with petroleum ether/Et_2_ O-gradient led to 1-acetyl-*β*-carboline (5 mg) and perlolyrin (3 mg) as two pale yellow solids. On subjecting the middle polar fraction (III) to further purification on silica gel (0.014-0.040 μm, 0.8×50 cm) using n-hexane/ethyl acetate, a crude white solid substance was obtained. Re-crystallization of the substance from *n*-hexane/ethyl acetate and from chloroform, respectively, yielded 12 mg of erythromycin A (1) as a white solid. Finally, purification of the high polar fraction V by silica gel (0.014-0.040 μm, 0.8x50 cm) using a DCM/MeOH gradient followed by Sephadex LH-20 (DCM/40%MeOH) delivered 44 mg HM1 as an oily substance.

### RESULTS AND DISCUSSION

Mutation was used as a major tool for the induction of a wide range of genetic variations for selection of higher antibiotic producers of marine *streptomycetes*. After treatment of the marine *Streptomyces* sp. AH2 with UV irradiation for different exposure times; 5, 10, 15 min, respectively, 145 isolates were obtained, and their productivity of bioactive compounds was examined.

Only 17, of all examined isolates were confirmed to produce higher (>10%) bioactivity than the original strain (AH2) (Table 1). Five (3.5%), nine (6.2%) and three (2.1%) mutants were isolated after treatment with UV light for 5, 10 and 15 min, respectively. It is obvious that a 10 min UV exposure time yields a higher number of mutants, with higher productivity of antibiotics than the parental strain. Three mutants (5/2, 10/9 and 10/14) were found to deliver 83.3% higher antibiotic activity against *E. coli* than the original strain, while four mutants 5/2, 10/9, 10/14 and 15/17 produced ~62.5% more antibiotic productivity than the original strain against *B. subtilis*.** Seven mutants (5/2, 5/25, 10/9, 10/14, 10/15, 15/17 and 15/19) were observed to produce ~55.6% higher antibiotic activity against *M. luteus*. (Table 1)

**TABLE 1:** TABLE 1: ANTIBIOTIC PRODUCTION CAPACITIES FOR MARINE *STREPTOMYCES* SP. INDUCED MUTANTS COMPARED WITH THE ORIGINAL STRAIN (AH2)

Isolate	Antibiotic productivity								
	
	*E. coli*			*B. subtilis*			*M. luteus*		
			
	mm*	μg/ml†	% versus W.T.	mm*	μg/ml†	% versus W.T.	mm*	μg/ml†	% versus W.T.
W.T.	16	300	100.0	22	400	100.0	25	450	100.0
5/2	22	550	183.3	30	650	162.5	30	700	155.6
5/6	18	400	133.3	25	500	125.0	27	525	116.7
5/8	18	400	133.3	28	575	143.8	28	600	133.3
5/15	17	350	116.7	26	525	131.3	28	600	133.3
5/25	20	500	166.7	29	600	150.0	30	700	155.6
10/3	18	400	133.3	26	525	131.3	28	600	133.3
10/8	19	450	150.0	28	575	143.8	29	650	144.4
10/9	22	550	183.3	30	650	162.5	30	700	155.6
10/11	18	400	133.3	26	525	131.3	28	600	133.3
10/12	17	350	116.7	25	500	125.0	28	600	133.3
10/14	22	550	183.3	32	700	175.0	33	750	166.7
10/15	20	500	166.7	29	600	150.0	30	700	155.6
10/19	19	450	150.0	28	575	143.8	29	650	144.4
10/22	18	400	133.3	26	525	131.3	26	500	111.1
15/17	20	500	166.7	30	650	162.5	30	700	155.6
15/11	19	450	150.0	28	575	143.8	29	650	144.4
15/19	20	500	166.7	29	600	150.0	30	700	155.6

* Inhibition zone diameter

† Antibiotic production according to erythromycin in standard cure

From the above study, the mutations of the marine *Streptomyces* sp AH2 genome by UV treatment, affected the production of antibiotics qualitatively and quantitatively. Chromatographic analysis of extracts of the selected 17 mutants using TLC in comparison with those of AH2, established that mutant 10/14 was the only one, which displayed more bands than the original one, and it was the most antibiotically active strain against all tested bacteria.

Chemical screening of the mutant 10/14 extract using TLC monitoring exhibited two major bands, which were not present in extracts of the parent strain. The first of them was Polar with no UV absorbance, which turned brown by spraying with anisaldehyde/sulphuric acid, and later to green. The other one showed an intensively blue UV fluorescence polar band, which turned yellow by anisaldehyde/sulphuric acid after heating.

Fermentation of a 20 L shaker culture using TSB medium at 28° was continued for 5 d. The culture broth was separated by centrifugation and extraction with ethyl acetate at pH 4. According to TLC monitoring, the mycelial cake did not contain interesting compounds and was discarded. The extract of filtrate was concentrated in *vacuo* followed by washing with brine and drying with anhydrous sodium sulphate. Finally, it was evaporated to dryness yielding a sticky brown extract.

Structures of the known compounds, phenazine^16^, 1-acetyl-*β*-carboline^17^ and perlolyrin^18^ were established on the bases of their chromatographic properties, NMR and MS spectroscopy as well as comparison with authentic spectra^19^.

Erythromycin A (1), a colourless solid, showed no UV absorbance (254 nm) or fluorescence (366 nm), however, it exhibited a brown colour by spraying with anisaldehyde/sulphuric acid, which turned later to green. The UV spectra displayed a peak at λ_max_ ~ 280, which is slightly bathochromic shifted in acidic solution to λ_max_ 289 nm. The IR spectra of 1 displayed an absorption band at ν 3450 cm^-1^ (hydroxyl/amino groups). Two strong bands at ν 1740 and 1720 are indicative for carbonyls of ester and ketone systems. It was lacking olefinic or aromatic signals, but displayed two strong bands between ν 1480~1340 cm^-1^ of methyl and methylene groups (Table 2).

**TABLE 2:** PHYSICO-CHEMICAL PROPERTIES OF ERYTHROMYCIN A (1)

	Erythromycin A (1)
Appearance	Colourless solid
*R_f_*	0.61‡
Molecular formula	C_37_H_68_NO_13_
(+)-ESI-MS: m/z (%)	734.3 [M^+^H]^+^ (100), 756.4 [M^+^Na]^+^, (12), 1489.3 [2M^+^Na], (32)
(-)-ESI-MS: m/z (%)	778.3 [M+HCOO]-
(+)-HRESI-MS	
Found	734.46835 (M+H; C_37_H_68_NO_13_)
Calc.	734.46850 (M+H; C_37_H_68_NO_13_)
IR (KBr)	3450, 2950, 1740, 1720, 1480, 1450, 1440, 1380, 1340, 1250, 1050
UV/VIS λ_max_	282 (ε 30, EtOH), 278 (ε 27, MeOH), 289 (ε 25.7, pH 6 buffer)
(α)^25^_D_(EtOH)	-78 (c, 1.99)

‡ EtOAC/MeOH/ACOH/H_2_O (3:3:0:5:0:5)

ESI MS of 1 showed three quasi molecular ion peaks at *m/z* 734.3 (M+H), 756.4 (M+Na) and 1489.3 (2M+Na) in the (+)-ESI-MS mode, and one at *m/z* 778.3 (M+HCOO^-^ ) in the (-)-ESI-MS mode. This established the molecular weight as 733 Dalton, which is indicative of the existence of an odd number of nitrogen atoms. HRESI MS of 1 [*m/z* 734.4683850, (M+H)] established the molecular formula as C_37_ H_67_ NO_13_, bearing 5 double bond equivalents. Compound 1 was subjected to detailed ESI-MS^2^ and MS^3^ fragmentations combined with HRESI-MS, from which, 9 and 11 peak-fragments, respectively, were assigned (Table 3).

**TABLE 3:** ESI-MS^2^ AND ESI-MS^3^ FRAGMENTATIONS OF ERYTHROMYCIN A (1)

HRESI-MS^2^- *quasi* ion fragments (MeOH+NH_4_OAc)	Formula	HRESI-MS^3^- *quasi* ion fragments (MeOH/H_2_O+Facid)	Formula
576.37487	C_29_H_54_NO_10_	716.45777 (M-H_2_O)	C_37_H_66_NO_12_
558.36408	C_29_H_52_NO_9_	698.44724 (M-2H_2_O)	C_37_H_64_NO_11_
540.35349	C_29_H_50_NO_8_	576.37484 (M-[3-methoxy-mycarose-H])	C_29_H_54_NO_10_
522.34292	C_29_H_48_NO_7_	558.36434	C_29_H_52_NO_9_
464.30119	C_26_H_42_NO_6_	540.35387	C_29_H_50_NO_8_
408.27500	C_23_H_38_NO_5_	522.34329 (M-[3-methoxy-mycarose-H]-3H_2_O)	C_29_H_48_NO_7_
342.22805	C_18_H_32_NO_5_	464.30176	C_26_H_42_NO_6_
233.15394	C_15_H_21_O_2_	408.27500	C_23_H_38_NO_5_
158.11770	C_8_H_16_NO_2_	342.22863	C_18_H_32_NO_5_
		233.15369	C_15_H_21_O_2_
		158.11778	C_8_H_16_NO_2_

**Fig. 1:**
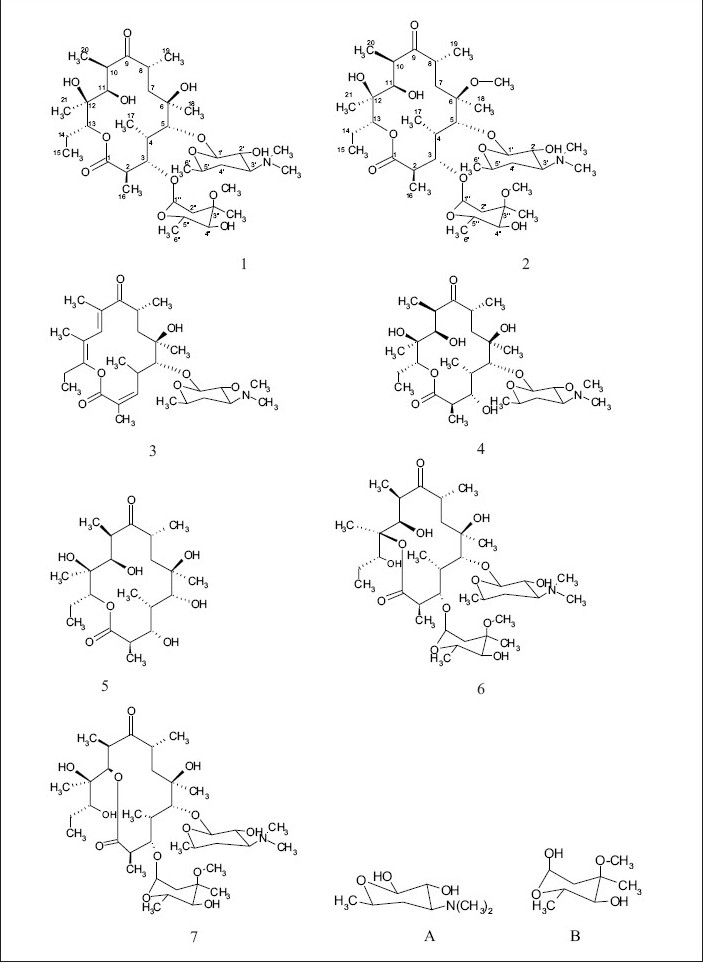
Structural formula of the macrolides 1-7 beside to the sugars A and B.

The common base peak fragment in both cases at *m/z* 158.1177 (C_8_H_16_NO_2_) was attributed to desosamine (A), and the later was further established by the characteristic N(CH_3_)_2_ signal at δ 2.38 in the ^1^H NMR spectrum. On the other hand, the neutral sugar, 3-methoxy-mycarose (B) was established by the existence of a typical ion at *m/z* 576.37484 (C_29_H_54_NO_10_ M-[3-methoxy-mycarose-H]), beside a characteristic methyl ether signal in the NMR spectrum (δ_H_ 3.26 and δ_C_ 49.4). The conjugated macrolide system still containing the desosamine moiety (3, *m/z* 522.34329 (C_29_ H_48_ NO_7_)) afforded after a consecutive loss of water the fragment ions at *m/z* 716.45777 (M-H_2_O), 698.44724 (M-2H_2_O), followed by the expulsion of mycarose sugar moiety (fig. 2a,b). Compound 3 is not known from nature or synthetic chemistry, while 5-O-desosaminylerythronolid A (4) does naturally exist (fig. 1).

**Fig. 2a:**
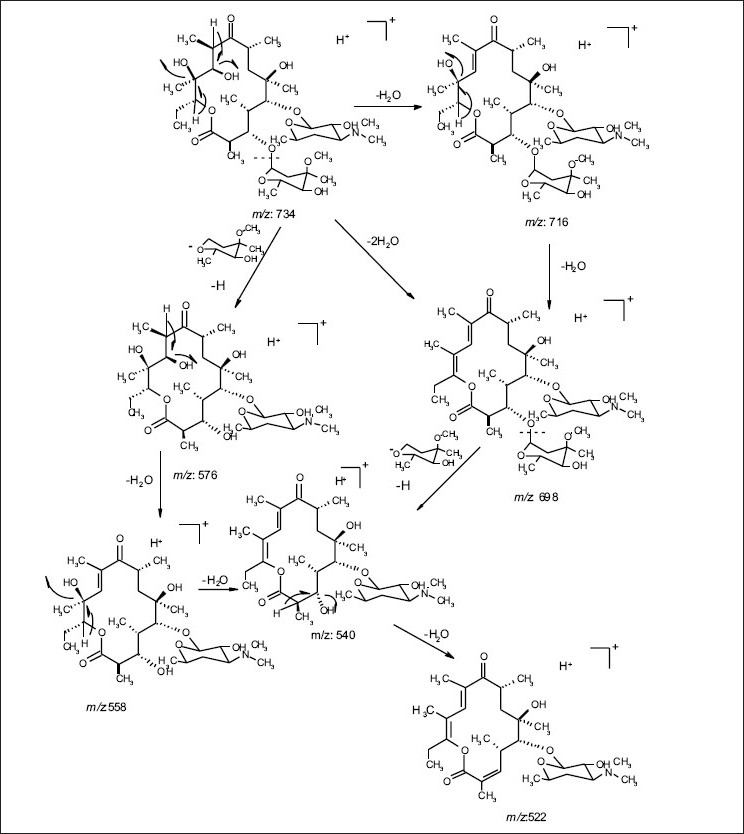
ESI-MS-MS fragmentations of erythromycin A(1)

**Fig. 2b:**
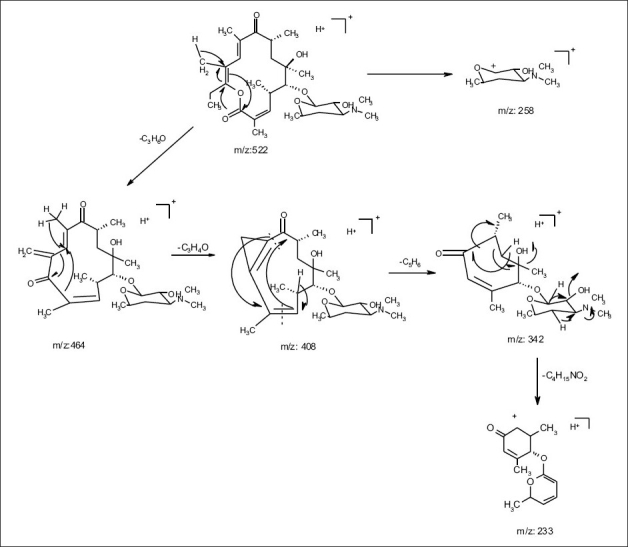
(+)-ESI-MS-MS fragmentations of erythromycin A (1)

It has been shown^20^,^21^ that erythromycins are assembled in *Sac. erythraea* from propionate and methyl malonate units initially forming the ‘aglycone’ 6-deoxyerythronolide B (6-dEB), which is then further oxidised by cytochrome P-450s and glycosylated to yield erythromycin A^22^. The biosynthesis of erythromycins may proceed through a pathway involving compound 4^23^, as it has been demonstrated, contrary to the prior held beliefs, that erythronolide A (5) is glycosylated to erythromycin A (1), and thus erythronolide A (5) might be not present in a free form (fig. 1).

Under EI MS conditions, the erythromycins displayed three characteristic ions at *m/z* 382 of the α γ unsaturated system of erythronolide A (5)^24^ (as a result of expulsion of the sugar moieties followed by elimination of 2H_2_O from C-2~C4), *m/z* 159 (cladinosyl, B) and finally a base peak at *m/z* 158 of the desosaminyl moiety (A). The lactone ring displayed also a fragmentation because of elimination of the C_12_-C_15_ fragment confirming the nature of the terminal methyl group bonded to C-15. Moreover, the neutral sugar was established based on two characteristic fragmentation peaks at *m/z* 127 and 115^25^.

In the ^1^H NMR spectra, compound 1 exhibited 6 methyl doublets (δ 1.25~1.12 ppm), 3 methyl singlets (δ 1.43~1.11 ppm), one methyl triplet (δ 0.84 ppm), one methoxy singlet (δ 3.26 ppm) and one N-(CH_3_ )_2_ singlet (δ 2.38 ppm). Two of the ten oxymethine protons (δ 5.05~2.95 ppm) were anomeric (δ 4.84 & 4.39 ppm), four were in the range of δ 3.05~2.66. Finally, it showed four methylene multiplets (δ 1.88~1.41 ppm).

**Fig. 3:**
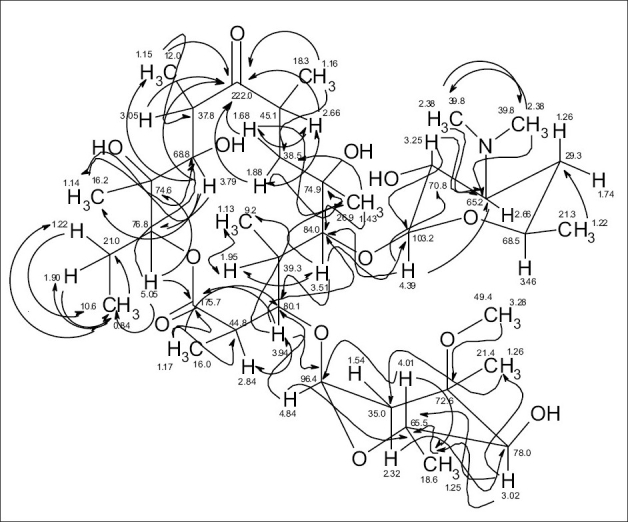
HMBC (→) and ^1^H, ^1^H COSY (↔) derived connectivities of erythromycin A (1)

The ^13^C NMR and HSQC spectra (Table 4) indicated 36 signals attributed to 37 carbons as demanded by the molecular formula. It depicted two carbonyl carbons, one of an ester (175.7 ppm) and the other one of a ketone system (222.0 ppm). The spectrum contained 13 methyl carbons, among them one methoxy and two equivalent methyls of the N-(CH_3_)_2_ moiety, 15 methine carbons, ten of them were oxygenated, and 4 methylene signals. Finally, 3 signals of quaternary sp^3^ carbons were visible. By careful interpretation of the 1D and 2D NMR data (fig. 3) in combination with the molecular formula and ESI-MS^2^ and ESI-MS^3^ fragmentations and comparison with related analogous^23^,^26^ (e.g. 6-O-methylerythromycin A (2)^27^, the structure of erythromycin A (1) was fully deduced, excluding also the initially suggested two macro-lactones with 13- and 12-memberd rings (6,7 fig. 1). This was confirmed by the direct cross-signal from the oxymethine proton at δ 5.05 ppm (H-13) towards the lactone carbonyl (C-1, 175.7 ppm) in the HMBC spectrum establishing the ring closure between C-13 and C-1 *via* oxygen, excluding structure 6. Moreover, the methine proton at δ 3.79 ppm (H-11) exhibited no correlation to the same lactone carbonyl, negating also structure 7.

**TABLE 4:** ^13^C NMR AND ^1^H NMR DATA OF ERYTHROMYCIN A(1) COMPARED WITH 6-O-METHYLERYTHROMYCIN A (2)

Position	Erythromycin A (1)		6-O-Methylerythromycin A (2)	
		
	*δ_C_* ppm	*δ_H_* ppm (*J* in Hz)	*δ_C_* ppm	*δ_H_* ppm
1	175.7	-	175.9	-
2	44.8	2.84 (dq, 10.0, 7.3)	45.1	2.89 (dq)
3	80.1	3.94 (dd, 10.0, 9.9)	78.5	3.77 (dd)
4	39.3	1.95 (ddq, 7.5, 7.3)	39.3	1.92 (ddq)
5	84.0	3.51 (d, 7.2)	80.8	3.67 (d)
6	74.96	-	78.5	-
7	38.5	1.88 (dd, 14.8, 7.8), 1.68 (dd, 14.8, 7.8)	39.4	1.72 (dd), 1.85 (dd)
8	45.1	2.66 (ddq, 7.7, 6.9)	45.3	2.59 (ddq)
9	222.0	-	221.1	-
10	37.76	3.05 (brq, 6.8)	37.3	3.00 (dq)
11	68.85	3.59 (brd, 9.8)	69.1	3.76 (d)
12	74.6	-	74.3	-
13	76.84	5.05 (dd, 9.9, 4.2)	76.7	5.05 (dd)
14	21.0	1.90 (ddq, 14.1, 10.1, 7.3 ), 1.22 (ddq, 14.1, 10.1, 7.3)	21.1	1.48 (ddq), 1.92 (ddq)
15	10.6	0.84 (t, 7.3)	10.6	0.85 (t)
16	16.0	1.17 (d, 7.3)	16.0	1.20 (d)
17	9.2	1.13 (d, 7.0)	9.1	1.10 (d)
18	26.9	1.43 (s)	19.8	1.41 (s)
19	18.3	1.16 (d, 6.9)	18.0	1.14 (d)
20	12.0	1.15 (d, 6.8)	12.3	1.13 (d)
21	16.2	1.14 (s)	16.0	1.12 (s)
6-OCH_3_	-	-	50.7	3.04 (s)
1′	103.3	4.39 (d, 7.2)	102.9	4.44 (d)
2′	70.8	3.25 (dd, 10.1, 7.2)	71.0	3.19 (dd)
3′	65.2	2.66 (ddd, 12.5, 10.1, 3.9)	65.6	2.41 (ddd)
4′	29.3	1.74 (ddd, 12.5, 12.5, 10.1 ), 1.26 (ddd, 12.5, 12.5, 10.1)	28.6	1.21 (ddd), 1.66 (ddd)
5′	68.5	3.46 (ddq, 10.1, 7.3 6.0)	68.8	3.48 (ddq)
6′	21.3	1.22 (d, 6.0)	21.5	1.23 (d)
3′-N(CH_3_)_2_	39.8	2.38 (s)	40.3	2.28 (s)
1″	96.4	4.84 (dd, 5.0, 1.2)	96.1	4.93 (dd)
2″	35.0	1.54 (dd, 15.0, 10.1), 2.32 (dd, 15.0, 10.1)	34.9	1.59 (ax, dd), 2.37 (eq, dd)
3″	72.6	-	72.7	-
4″	78.0	3.02 (dd, 9.4, 3.1)	78.0	3.02 (dd)
5″	65.46	4.01 (dq, 9.4, 6.1)	65.8	4.01 (dq)
6″	18.6	1.25 (d, 6.1)	18.7	1.30 (d)
7″(3″-CH_3_)	21.4	1.26 (s)	21.5	1.25 (s)
3″-OCH_3_	49.4	3.28 (s)	49.5	3.33 (s)

The aglycone, erythronolide A (5) and more than 40 derivatives are known from nature^28^. Erythromycin derivatives are well known with potent activity against Gram-positive bacteria, most of them showing additional DNA binding properties^29^. Recently, erthromycin A (1) was found to be of therapeutic use for treatment of inflammatory immuneoreactions that may be the major cause of morbidity or mortality associated with “Bird Flue” influenza infection^30^.
